# Enhancing Orange Juice Shelf Life Using Soy Lecithin‐Stabilized Lemongrass Essential Oil Nanoemulsions: Formulation, Optimization, and Application

**DOI:** 10.1002/fsn3.70398

**Published:** 2025-06-19

**Authors:** Mahsa Nouraddini, Forogh Mohtarami, Mohsen Esmaiili

**Affiliations:** ^1^ Food Science and Technology Department, Agricultural Faculty Urmia University Urmia Iran

**Keywords:** antimicrobial activity, encapsulation efficiency, gastrointestinal condition, grape seed oil, release behavior

## Abstract

In this study, a nanoemulsion of lemongrass essential oil was prepared using a formulation comprising lemongrass essential oil (1%–10%), soy lecithin (1%–10%), and water (80%–98%) via ultrasonication. The optimal formulation was selected based on its lowest polydispersity index (0.27) and particle size (187.01 nm), alongside its highest zeta potential (−28.34 mV) and encapsulation efficiency (88.20%). The effects of grape seed oil (0%, 1.5%, and 3% w/w) and Tween 80 (0%, 1.5%, and 3% w/w) on the characteristics of the optimal lemongrass essential oil–lecithin formulation were then evaluated. Based on the same extrema, two optimal samples were selected: one with 6% lemongrass essential oil–lecithin (without grape seed oil or Tween 80) and another with 6% lemongrass essential oil–lecithin, 1.5% grape seed oil, and 1.5% Tween 80. The nanoemulsions were characterized using scanning electron microscopy and Fourier transform infrared spectroscopy, and their release profiles, antimicrobial activity, and antioxidant properties were assessed. When the nanoemulsions (0%, 0.2%, 0.4%, and 0.6% w/w) incorporated into orange juice, enhanced antioxidant activity (from 14.38% to 77.38%) and reduced bacterial counts (from 5.13 to 4.10 log CFU/mL at 0.6% concentration). Sensory evaluation after 15 days at 4°C revealed improved taste, aroma, and overall acceptance, with the highest scores for the 0.2% lemongrass essential oil–lecithin sample (without grape seed oil or Tween 80). This method effectively masks essential oil flavors while boosting the juice's nutritional value, stability, and health benefits.

## Graphical abstract

In this study, lemongrass essential oil (LGEO) nanoemulsions were successfully developed using soy lecithin and ultrasonication, optimizing stability and bioactivity. Grape seed oil (GSO) and Tween 80 were then integrated to enhance the nanoemulsion properties, achieving a balance between encapsulation efficiency and particle size. The optimized nanoemulsion demonstrated strong inhibitory effects against microbial and enzymatic spoilage in orange juice. Finally, the incorporation of LGEO nanoemulsions preserved the quality of the orange juice, extending its shelf life while maintaining flavor and nutritional integrity.

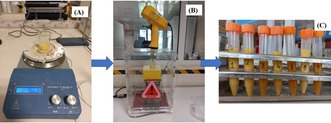

## 1 Introduction

Nowadays, worldwide interest in consuming nutritious and natural foods has increased. Natural fruit juices are favored among consumers due to their excellent nutritional value and pleasant taste. Oranges, from the *Rutaceae* family, have a desirable flavor and color, along with high nutritional value, making orange juice one of the most consumed fruit beverages. Oranges are rich in minerals, vitamins (A, B, and C), and important flavonoids such as hesperidin, naringin, neo‐hesperidin, and rutin. Orange juice, with its attractive color, fresh taste, and high nutritional value, is a popular beverage enjoyed globally (Tütem et al. 2020; Wibowo et al. 2015). Fruit juice is subject to degradation due to factors such as microbial spoilage, enzymatic activity, or a combination of both. Methods such as thermal pasteurization, freezing, and the addition of antimicrobial compounds have been used to extend the shelf life of beverages (Franklyne et al. 2019; Napiórkowska et al. 2024); however, these methods may slightly alter the flavor and nutritional content compared to freshly squeezed juice. One alternative is the incorporation of natural compounds with antimicrobial and antioxidant properties, such as essential oils (EOs), into food products. These EOs effectively prevent microbial spoilage and increase shelf life (Liu et al. 2021), without compromising nutritional value. Numerous studies highlight lemongrass (LG) and its EO for their antifungal and antibacterial activities against a wide range of microorganisms (Antonioli et al. 2020; Fokom et al. 2019; Mukarram et al. 2021). LG ( *Cymbopogon citratus* ), from the *Poaceae* family, is rich in vitamins A, C, E, folate, niacin, riboflavin, proteins, antioxidants, and essential minerals such as nitrogen, phosphorus, potassium, magnesium, zinc, sulfur, manganese, iron, and copper, making it valuable for both human and animal consumption (Mukarram et al. 2021). However, the EO's volatile compounds limit its use in food products due to challenges like strong aroma, low stability, high volatility, and sensitivity to environmental conditions. To address these limitations, nanoemulsions (NEs) can encapsulate EOs, reducing the required quantities, enhancing stability, minimizing undesirable flavors, and improving distribution in food products (Bento et al. 2020; Sharma et al. 2022).

Among encapsulation methods for bioactive ingredients, NEs are highly suitable for food applications. NEs consist of two immiscible phases—a continuous phase and a dispersed phase—with particle diameters of 10–100 nm. Their small size offers advantages such as enhanced physicochemical stability, controlled EO release, increased clarity, reduced phase separation, and improved bioactivity (Hosseiniyeh et al. 2024). NE fabrication employs low‐energy (e.g., spontaneous emulsification) or high‐energy (e.g., ultrasonication, high‐pressure homogenization) methods, influencing stability (Bagheri et al. 2020; Sugumar et al. 2016). Although thermodynamically unstable, NEs are kinetically stable and differ from conventional emulsions in particle size (Akkam et al. 2021). Ultrasonication is particularly favored for its cost‐effectiveness and simplicity (Perumal et al. 2021).

Studies have explored NE formulations for various EOs, including orange oil (Asadinezhad et al. 2019), thyme (Jabraeili et al. 2022), bergamot (Li et al. 2019), and oregano (Zhao et al. 2023). Applications in food products include thyme essential oil nanoemulsions (EONEs) in ultrafiltered cheese (El‐Sayed and El‐Sayed 2021), *Thymus daenensis* EONEs in mayonnaise (Mansouri et al. 2021), cinnamon EONEs in apple juice (Xu et al. 2020), and antimicrobial eugenol nanoemulsions in fruit juices (Ghosh et al. 2014).

In response to the growing consumer preference for clean‐label and natural food products, this study aims to develop a robust nanoemulsion of lemongrass essential oil (LGEO) to significantly enhance the quality, safety, and shelf life of orange juice. By leveraging advanced NE technology, we seek to effectively encapsulate LGEO, reducing its strong sensory impact while utilizing its potent antimicrobial and antioxidant properties. This research strives to bridge the gap between the functional benefits of EOs and their practical application in food systems, offering a sustainable solution for food preservation.

## 2 Materials and Methods

### 2.1 Materials

Dried mature leaves of the LG plant and orange fruits were obtained from a local market in Urmia, West Azerbaijan Province, Iran. Soy lecithin (purity > 97%, CAS No. 8002‐43‐5) and a dialysis bag (20 mm diameter, 12 kDa, 99.99% retention; Art. No. D0530) were purchased from Sigma‐Aldrich (USA). GSO (CAS No. 6439‐56‐5) was acquired from Loba Chemie (India).

*Staphylococcus aureus* (PTCC 1112) and *Escherichia coli* O157:H7 (PTCC 133) were procured from the Iranian Industrial Fungi and Bacteria Collection Center. Nutrient agar, potato glucose agar, Tween 80, phosphate‐buffered saline (PBS), glycerol, and other chemical reagents were supplied by Merck (Germany).

### 2.2 Extraction of LGEO and Determination of Its Components

The LGEO was extracted via hydrodistillation using a Clevenger apparatus. Dried LG leaves were ground with an electric grinder (Bosch, BSH2616, Germany) and mixed with water at a 1:5 (w/v) ratio in the distillation flask. For chemical characterization, LGEO components were analyzed by gas chromatography–mass spectrometry (GC/MS) (Waltham, MA, USA) equipped with an HP‐5MS column (30 m × 0.25 mm ID, 0.25 μm film thickness). The oven temperature was programmed from 50°C to 280°C at a rate of 2°C/min (Fokom et al. 2019).

### 2.3 Preparation of Nanoemulsions

**FIGURE 1:**
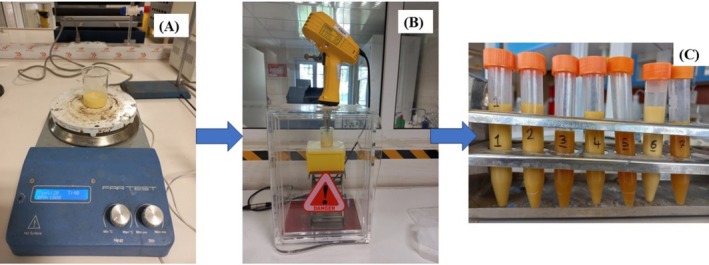
Preparation of LGEO nanoemulsion (A) mixture of raw material by a magnetic stirrer, (B) ultrasonication of NE, and (C) prepared NE samples.

The oil‐in‐water NE of LGEO was prepared at room temperature using the ultrasonic emulsification method (Yazgan 2020). Initially, varying ratios of LGEO (1%–10%), soy lecithin (1%–10%), and water (80%–98%) were combined according to a mixture design (Table 1) and homogenized for 10 min using a magnetic stirrer (Labinco L82, Netherlands). The pre‐emulsion was then sonicated (UP200Ht, Hielscher, Germany) at 200 W and 20 kHz for 10 min. To minimize heat generation during sonication, the sample was maintained in an ice bath (Figure 1).

**TABLE 1:** Mixture design for the preparation of LGEO nanoemulsion.

Run	Water (%)	EO (%)	SL (%)
1	88.2	5.9	5.9
2	82.4	8.8	8.8
3	80.0	10.0	10.0
4	91.6	4.2	4.2
5	98.0	1.0	1.0
6	95.0	2.5	2.5
7	87.2	6.4	6.4
8	98.0	1.0	1.0
9	84.4	7.8	7.8
10	80.0	10.0	10.0
11	87.2	6.4	6.4
12	93.2	3.4	3.4
13	95.0	2.5	2.5

The optimal sample from the initial stage was selected based on encapsulation efficiency (EE), zeta potential, particle size, and polydispersity index (PDI) using numerical optimization. This optimized formulation served as the base to evaluate the effects of GSO (0%, 1.5%, and 3% w/w) and Tween 80 (0%, 1.5%, and 3% w/w) as a co‐emulsifier on the NE properties (Table 2). For preparation, soy lecithin was first dissolved in water under magnetic stirring (30 min). LGEO (6%) was then gradually incorporated, followed by grape seed oil (GSO) and Tween 80 after 5 min. The mixture was stirred for 2 h and subsequently sonicated (200 W, 20 kHz, 10 min) (Liew et al. 2020). The optimal formulation from the second stage (selected based on EE, zeta potential, particle size, and PDI) was added to orange juice at varying concentrations.

**TABLE 2:** Completely randomized design for the preparation of the second stage NE.

Run	EO (%)	SL (%)	GSO (%)	Tween 80 (%)
1	6	6	0	0
2	6	6	1.5	1.5
3	6	6	1.5	3
4	6	6	3	1.5
5	6	6	3	3
6	6	6	3	0
7	6	6	0	3

### 2.4 Zeta Potential and Particle Size

The zeta potential, particle size, and particle size distribution of the NE sample of LGEO, derived from the first and second stages, were studied using dynamic light scattering (DLS) with a Zetasizer (ZEN360, Malvern Instruments Ltd., United Kingdom Nano ZS) (Hosseiniyeh et al. 2024).

### 2.5 Encapsulation Efficiency (EE)

The EE is defined as the ratio of the EO encapsulated within the wall materials to the total EO present in the microparticles (both free and encapsulated EO). To determine the EE of the NE samples, approximately 0.01 ± 0.001 g of the sample was weighed and dissolved in 15 ± 0.01 mL of distilled water, then centrifuged for 1 min at 8000× *g*. The supernatant was collected, and its absorbance was read at 240 nm (nm), indicating the amount of free EO. Subsequently, the samples were centrifuged again for 40 min, and the absorbance of the supernatant at 240 nm was measured, reflecting the total EO content (Karimi Sani et al. 2019). The calibration curve for LGEO was also plotted. Finally, the EE was calculated using the following equation:

(1) $$\mathrm{EE}\left(\%\right)=\frac{\text{Total}\;\mathrm{EO}-\text{Free}\;\mathrm{EO}}{\text{Total}\;\mathrm{EO}}\times 100$$

### 2.6 Scanning Electron Microscopy (SEM)

The surface morphology and microstructure of the optimum NE particles were imaged using SEM (Tescan, MIRA III, Czech Republic). A film was prepared by depositing the sample onto a carbon‐coated copper grid. Excess sample was removed, and the film was dried under a mercury lamp for 5 min before SEM imaging (Sundararajan et al. 2018).

### 2.7 Fourier Transform Infrared (FTIR) Spectroscopy

The absorption spectra of the NE samples were recorded at a wavenumber range of 4000–400 cm^−1^ using an FTIR spectrophotometer (System 200, Perkin Elmer, Wellesly, MD, USA). For this purpose, the samples were prepared as potassium bromide disks. Specifically, 2 ± 0.001 mg of the dried NE samples were mixed with potassium bromide to form the disks (Ranjbar et al. 2023).

### 2.8 Release Behavior

The release of EO from the NEs under simulated gastrointestinal conditions was evaluated using a dialysis bag according to the method of Hou et al. (2021) with some modifications. In the simulated stomach phase, 2 ± 0.01 mL of the NE sample was placed into a dialysis bag (12 kDa, diameter of 20 mm, Sigma, USA) and sealed on both ends. It was then immersed in 200 ± 0.01 mL of phosphate buffer with a pH of 7.4 ± 0.1. The phosphate buffer was maintained at 37°C ± 1°C while stirring at 100 ± 1 round per minute (RPM). At specific time intervals, the amount of released EO in the gastric and intestinal environments was measured at *λ*_max_ of LGEO (240 ± 1 nm) using a UV–VIS spectrophotometer (T60 UV, PG Instrument, Australia). For the preparation of simulated gastric fluid, 0.5 ± 0.01 g/L of pepsin was added to the phosphate buffer, and its pH was adjusted to 1.2 ± 0.1 with HCl. For the simulated intestinal fluid, 1 ± 0.01 g/L of pancreatin and 3 ± 0.001 g/L of bile salts were added to the phosphate buffer, and the pH was adjusted to 7.4 ± 0.1 with NaOH.

### 2.9 Antimicrobial Activity

The antimicrobial activity of the optimized NEs of LGEO was examined using the well diffusion method. In this method, plates containing Mueller–Hinton agar medium were inoculated with *Staphylococcus aureus* (1431 PTCC) and *Escherichia coli* H157:O7 (1399 PTCC). The growth inhibition zone was evaluated and reported as the antimicrobial activity (Gorzin et al. 2024).

### 2.10 Preparation of Orange Juice

**FIGURE 2:**
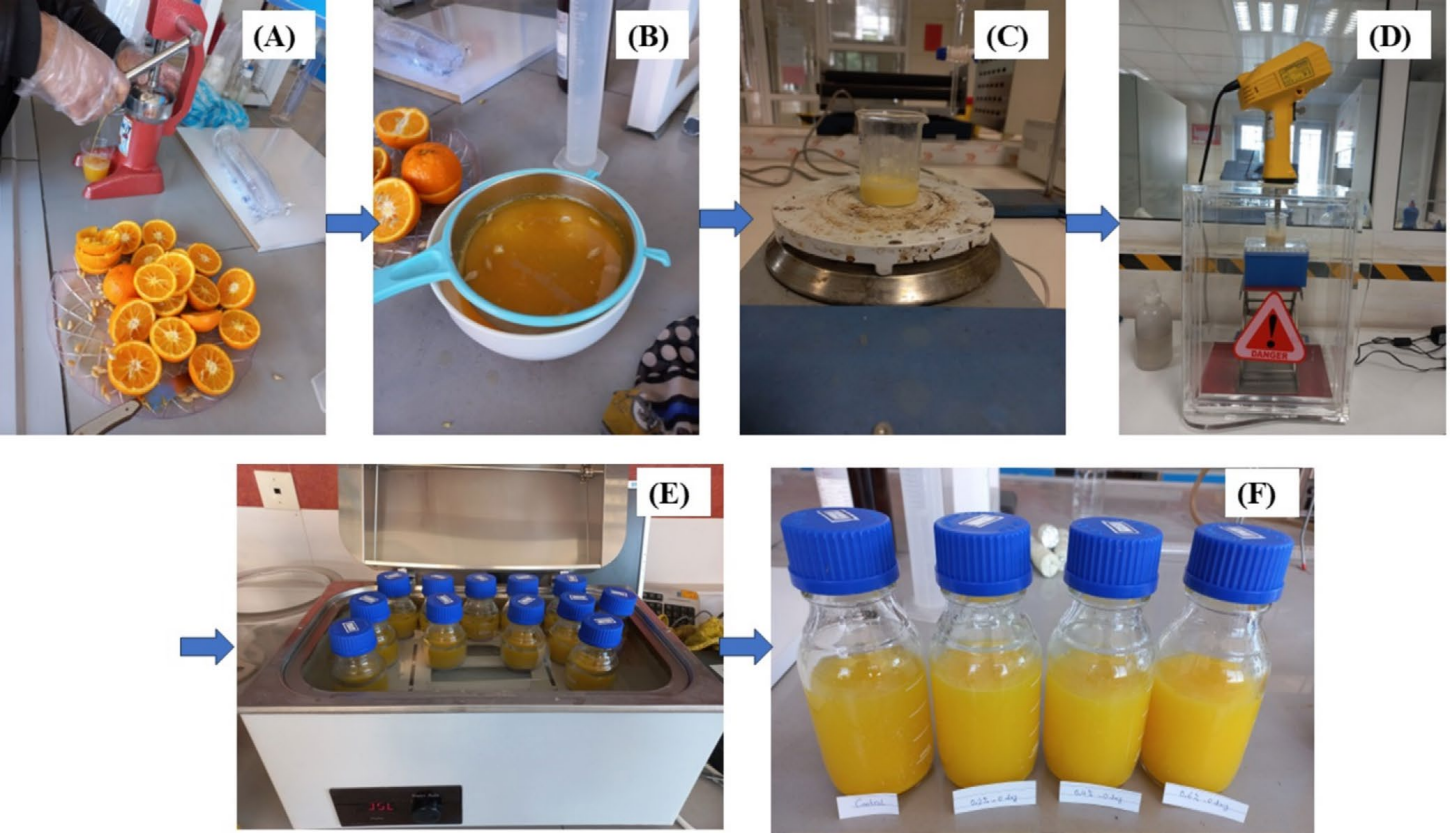
Preparation of orange juice containing LGEO nanoemulsion (A) juicing oranges, (B) filtering the orange juice, (C) mixing the orange juice with LGEO nanoemulsion, (D) ultrasonication of samples, (E) pasteurization of samples at water bath, and (F) prepared orange juice samples.

The oranges were washed with water after purchase, cut in half, and juiced using a manual juicer. The juice was then filtered through a double‐layer cloth to remove the pulp. The freshly extracted juice was poured into glass containers, and nano‐emulsified EO was added at varying concentrations (0%, 0.2%, 0.4%, and 0.6% w/w). The jars were tightly sealed and pasteurized in a water bath (WNB 22, Memmert, Germany) at 85°C ± 0.1°C for 15 min. Finally, the samples were refrigerated at 4°C ± 1°C until further analysis (Figure 2) (Ouazzou et al. 2025).

### 2.11 Characterization of Orange Juice Samples

#### 2.11.1 Acidity

To measure acidity, 10 ± 0.1 mL of the orange juice was diluted with 10 ± 0.1 mL of distilled water, and then ⁓4 drops of phenolphthalein indicator were added. Finally, the resulting solution was titrated with 0.1 N sodium hydroxide until a stable pink color (pH value above 8.2 ± 0.1) was reached (Cenobio‐Galindo et al. 2019).

#### 2.11.2 Antioxidant Activity

The antioxidant activity of NEs and the orange juice samples containing nano‐emulsified LGEO was assessed using the 2,2‐diphenyl‐1‐picrylhydrazyl (DPPH) radical scavenging activity test. For this purpose, 100 ± 0.01 μL of the sample was mixed with 2.9 ± 0.1 mL of a methanolic DPPH radical scavenging activity solution (0.1 mM). The resulting solution was well mixed for 5 min and then left in darkness for 60 min, after which its absorbance at a wavelength of 517 ± 1 nm was measured using a spectrophotometer (T60 UV, PG Instrument, Australia). Additionally, the control sample was prepared with 100 ± 0.01 μL of methanol and 2.9 ± 0.1 mL of DPPH (Xu et al. 2020). The DPPH radical scavenging activity was calculated using the following equation:

(2) $$\text{DPPH Radical scavenging activity}\;\left(\%\right)=\frac{A_{c}-A_{s}}{A_{c}}\times 100$$

where *A*_c_ = absorbance of the control and *A*_s_ = absorbance of the sample.

#### 2.11.3 Microbial Count

The total bacterial count, molds, and yeasts in orange juice were measured according to Zhou et al. (2021). In this method, 1 ± 0.1 mL of the diluted orange juice samples was poured into plates containing nutrient agar for bacterial counting and potato glucose agar for mold and yeast. The plates were incubated at 37°C ± 1°C for 48 h for total bacterial count and at 28°C ± 1°C for 5 days for mold and yeast counting. The number of colonies that grew was then counted and reported as log colony forming unit/mL (CFU/mL).

#### 2.11.4 Sensory Evaluation

Sensory characteristics (color, flavor, aroma, and overall acceptability) of the orange juice samples containing NE of LGEO during storage (0, 15, and 30 days) were judged by a panel of 15 semi‐trained panelists based on a 5‐point Hedonic scale ranging from 1 (very bad) to 5 (very good) (Radi et al. 2018).

### 2.12 Statistical Analysis

In the first stage, a D‐optimal mixture design was used, incorporating three formulation components: emulsifier (1%–10%), EO (1%–10%), and water (80%–98%). The emulsion was prepared at a 1:1 ratio of EO to emulsifier. In the second and third stages, a completely randomized design was employed.

The second stage analyzed the effect of formulation on NE results using one‐way ANOVA, while the third stage examined the influence of NE concentration and storage time on orange juice samples using two‐way ANOVA. Means were compared using Duncan's post hoc test, with significant differences determined at *p* < 0.05.

All experiments were performed in triplicate, and data are presented as mean ± standard deviation. Data analysis and graphing were conducted using Design‐Expert 7.0.0 and SPSS Statistics (version 26).

## 3 Results and Discussion

### 3.1 GC–MS Analysis of EO

The chemical composition analysis of LGEO, conducted using GC–MS, is presented in Table 3. The results reveal the presence of 23 bioactive compounds in the LGEO, with menthol being the predominant constituent (33.86%). Other primary compounds identified in LGEO included geraniol (18.46%), neral (10.75%), citral (7.37%), and β‐pinene (5.08%). Fokom et al. (2019) identified neral (34.76%) and geraniol (31.45%) as the major constituents of LGEO, while Antonioli et al. (2020) reported higher proportions of geraniol (41.8%) and neral (25.6%). These variations may result from differences in growth environments, climatic conditions, or plant species.

**TABLE 3:** Identified components in LGEO using GS‐MS analysis.

	Compounds	Area (%)	Rt. (min)
1	Menthol	33.68	7.132
2	Geraniol	18.46	5.089
3	Neral	10.75	15.533
4	Citral	7.37	16.149
5	β‐Pinene	7.36	10.428
6	Carane	5.08	14.223
7	Barnanone	3.11	13.834
8	Ethyl linalool	2.25	12.642
9	Acetate geranil	2.16	18.169
10	Decalin	2.15	13.59
11	Dicyandiamid	1.32	5.983
12	Naphthalene	1.30	29.496
13	Decan	1.23	10.003
14	Geranyl formate	1.09	4.396
15	Limonene	0.74	11.557
16	Menthyl acetat	0.50	17.797
17	2‐Methyl isoburneol	0.26	17.254
18	Farnesene	0.26	27.142
19	Zerumbone	0.24	20.551
20	Carvacrol	0.23	19.452
21	Caryophyllene oxide	0.18	23.848
22	Junipercamphor	0.17	20.077
23	Palmitic acid	0.11	22.583

### 3.2 Particle Size and PDI

**FIGURE 3:**
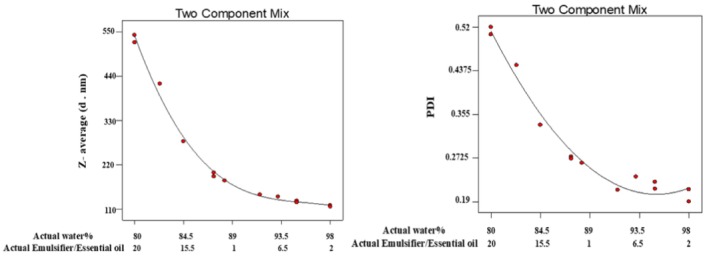
Effect of LGEO and SL on the *Z*‐average and PDI of EONE.

The formulation used in NE production critically influences key characteristics, particularly in achieving optimal particle sizes (20–200 nm) and low polydispersity (PDI < 0.5). Moreover, particle size serves as a fundamental determinant of product properties, significantly affecting appearance, bioavailability, stability, and texture (Mohammed et al. 2020). The PDI value expresses the particle size distribution, meaning a lower PDI indicates a narrower size distribution (Yazgan et al. 2019). In this study, NEs were prepared using varying concentrations of LGEO, SL emulsifier, and water. The particle size and PDI of the LGEO‐NEs are presented in Figure 3. The results demonstrated that the dispersed‐phase‐to‐emulsion ratio significantly influenced both particle size and PDI (*p* < 0.05). The NE samples exhibited particle sizes ranging from 125.8 to 541.2 nm. Notably, the formulation with the highest LGEO and emulsifier content yielded the largest particle size (541.2 nm) and highest PDI (0.536), whereas the sample with the lowest EO and emulsifier concentrations showed the smallest particle size (125.8 nm) and lowest PDI (0.287). This trend suggests that higher ratios of EO and emulsifier may promote particle coating, leading to less uniform and larger droplet formation (Mohammed et al. 2020). Furthermore, this observation can be attributed to the physicochemical properties of LGEO. Due to its partial water solubility, the oil phase tends to coalesce, forming larger droplets and consequently increasing the particle size of the NE (Liu et al. 2021). The increase in particle size with higher EO concentration may also result from Ostwald ripening, which modifies the oil–surfactant–water system dynamics in the given formulation. These findings align with previous reports by Liu et al. (2022) and Nirmal et al. (2018) for lemon EONE, who observed a similar trend of increasing particle size and PDI with elevated EO content.

### 3.3 Zeta Potential

**FIGURE 4:**
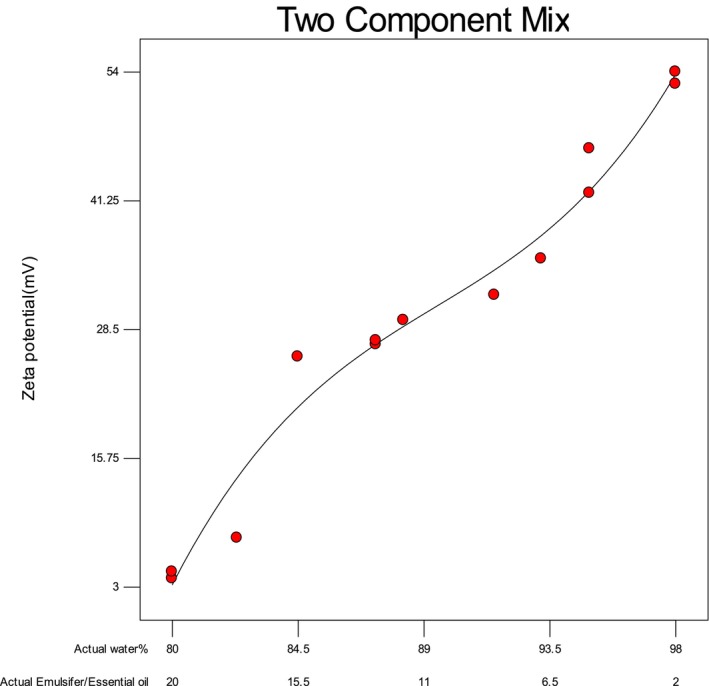
Effect of LGEO and SL on the zeta potential of EONE.

Zeta potential is a key indicator of electrostatic interactions between particles and the stability of NEs. It reflects the net surface charge of particles, where a higher absolute zeta potential indicates greater stability due to stronger electrostatic repulsion between droplets. Conversely, a lower absolute value suggests reduced stability, leading to droplet aggregation over time (Sahafi et al. 2018). The results (Figure 4) demonstrated that substituting LGEO and SL emulsifier with distilled water significantly affected the zeta potential of the NE samples (*p* < 0.05). All NE formulations exhibited negative zeta potential values ranging from −3.85 to −52.8 mV. This negative charge primarily originated from the SL emulsifier, which possesses anionic characteristics. Molet‐Rodríguez et al. (2021) reported zeta potential values ranging from −1.37 to −40.17 mV for NEs containing LG and orange EOs. In our study, progressive replacement of LGEO and SL emulsifier with distilled water resulted in a corresponding shift in zeta potential from −3 mV to −52 mV across the sample series.

The formulation with the highest concentration of EO and SL emulsifier demonstrated enhanced stability, correlating with its more negative zeta potential. This finding aligns with previous reports on black seed EONEs, which showed zeta potential values ranging from −14 to −31 mV (Mohammed et al. 2020).

### 3.4 Encapsulation Efficiency (EE)

**FIGURE 5:**
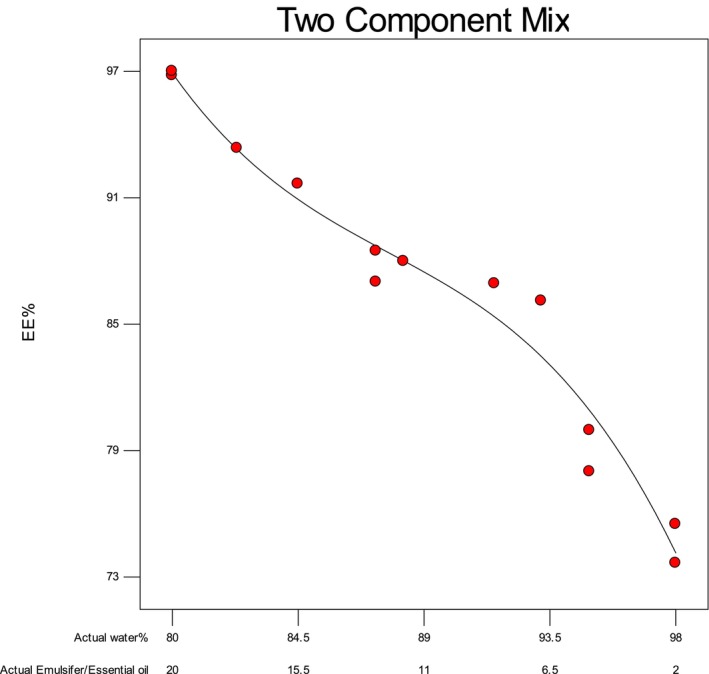
Effect of LGEO and SL on the encapsulation efficiency of the LGEO nanoemulsion.

The EE results for the LGEO nanoemulsion prepared with the SL emulsifier are shown in Figure 5. The EE of the NE samples ranged from 76.8% to 97%. At higher concentrations of EO and emulsifier, the EE reached its maximum (97%), whereas reducing the EO content to 2% decreased the EE to 73%. The high EE observed at elevated EO and emulsifier levels can be attributed to SL's lower molecular weight, which enhances molecular mobility and surface activity. This promotes the formation of a dense interfacial layer around the LGEO droplets, improving coverage (Hemmatkhah et al. 2020). Additionally, high EE minimizes EO migration to the particle surface, protecting it from oxidation and preserving its functional properties (e.g., antioxidant and antimicrobial activity) during storage. Hosseiniyeh et al. (2024) reported that the EE in the black seed oil NE increased with the addition of lecithin. Furthermore, Talón et al. (2019) also reported that the EE in the mint EONE prepared with lecithin was higher than that in the mint EONE with whey protein.

### 3.5 Optimal Formulation of Nanoemulsion (EO, Water, and Emulsifier Concentrations)

The optimal sample from the first stage of this study was selected based on the lowest PDI (0.27), highest zeta potential (−28.34 mV), and maximum EE (88.20%). The formulation containing 88% distilled water, 6% LGEO, and 6% SL emulsifier demonstrated the best performance, with a desirability score of 0.74. This sample was further investigated in the second stage by incorporating GSO and Tween 80.

### 3.6 Particle Size, PDI, and Zeta Potential

Table 4 shows significant differences in particle size and PDI among the LGEO nanoemulsion samples influenced by GSO and Tween 80 concentrations (*p* < 0.05). All prepared NEs exhibited particle sizes below 200 nm. The largest particles were observed in samples 1 (containing neither GSO nor Tween 80) and 6 (3% GSO without Tween 80), while the smallest size occurred in the sample with 3% GSO and 3% Tween 80. The results demonstrate that the addition of Tween 80 significantly reduced particle size, likely by preventing aggregation through enhanced adsorption at the water–oil interface and consequent surface tension reduction. This effect can be attributed to Tween 80's low molecular weight, which enables rapid coverage of newly formed droplets. Therefore, a sufficient amount of surfactant molecules must be present to cover the surface of the oil droplets during the formation of the NE (Artiga‐Artigas et al. 2018). Additionally, increasing the concentration of the emulsifier allows more emulsifier molecules to distribute from the oily phase to the aqueous phase, making it easier to produce smaller NE droplets (Nejatian and Abbasi 2019). The results are consistent with previous studies, where Artiga‐Artigas et al. (2018) observed that higher Tween 80 concentrations reduced particle size in LGEO nanoemulsions. Similarly, Hou et al. (2021) reported decreased particle sizes in cinnamon EONE upon the addition of Tween 80.

**TABLE 4:** Effect of LGEO, SL, GSO, and Tween 80 on the Z‐average, PDI, zeta potential, and EE of LGEO nanoemulsion.

Run	*Z*‐average (nm)	PDI	Zeta potential (mv)	Encapsulation efficiency (%)
1	159.51 ± 0.76^g^	0.26 ± 0.01^a^	−48.60 ± 0.18^a^	97.70 ± 0.85^f^
2	131.87 ± 0.64^e^	0.35 ± 0.00^c^	−41.44 ± 0.37^c^	89.55 ± 0.57^d^
3	64.08 ± 0.14^b^	0.33 ± 0.00^b^	−28.49 ± 0.41^f^	82.05 ± 0.38^b^
4	126.38 ± 0.17^d^	0.40 ± 0.00^d^	−37.61 ± 0.42^e^	80.90 ± 0.41^a^
5	61.69 ± 0.17^a^	0.33 ± 0.01^bc^	−21.51 ± 0.28^g^	81.28 ± 0.67^ab^
6	157.32 ± 0.36^f^	0.28 ± 0.00^a^	−46.03 ± 0.31^b^	84.28 ± 0.53^c^
7	81.74 ± 0.70^c^	0.31 ± 0.01^b^	−38.45 ± 0.30^d^	92.93 ± 0.32^e^

*Note:* Different small letters represent a significant difference (*p* ≤ 0.05) per column.

The lowest PDI was observed in the samples without GSO and in the NE sample with 3% GSO and 0% Tween 80. Conversely, the highest PDI was observed in the sample with 3% GSO and 1.5% Tween 80. Generally, the addition of Tween 80 as a co‐emulsifier led to an increase in the PDI of the samples. Asadinezhad et al. (2019) attributed the increased PDI following the addition of Tween 80 to elevated surface viscosity, which restricts emulsifier migration into the aqueous phase.

Both GSO and Tween 80 significantly influenced zeta potential values (*p* < 0.05) (Table 4). All formulations exhibited negative zeta potentials, with sample 1 (containing neither GSO nor Tween 80) showing the strongest negative charge (−48.60 ± 0.18 mV). In contrast, sample 5 (with 3% GSO and 3% Tween 80) displayed the weakest negative potential (−21.51 ± 0.28 mV). Notably, zeta potential magnitudes of ≥ |30| mV (either positive or negative) typically indicate enhanced NE stability. The negative charge on oil droplets likely originates from anionic hydroxyl groups present in the aqueous or oil phases of the NE (Hou et al. 2021). Anionic emulsifiers like SL (primarily phosphatidylcholine) can enhance this negative zeta potential due to their inherent negative charge. In contrast, non‐ionic Tween 80 cannot directly contribute to surface charge. The observed negative potential with Tween 80 may instead result from ionic impurities from production or storage, free fatty acids in oil/emulsifier components, or adsorption of negative ions by trace contaminants (Sahafi et al. 2021). Similar results have been reported by Kaur et al. (2021) for cinnamon EONE, where samples prepared with SL had a greater negative zeta potential compared to Tween 80. Zhao et al. (2023) also reported negative zeta potential values in the range of −15 to −44 mV for the oregano EONE with various emulsifiers.

### 3.7 Encapsulation Efficiency

The EE of LGEO nanoemulsions was significantly affected by GSO and Tween 80 concentrations (*p* < 0.05). Sample 1 (without GSO or Tween 80) showed the highest EE (97.70% ± 0.85%), while formulations containing 3% GSO with either 1.5% or 3% Tween 80 exhibited the lowest EE values (80.90% ± 0.41% and 81.28% ± 0.67%, respectively). This inverse relationship suggests that both additives reduce EE. The superior EE in sample 1 may be attributed to SL's favorable properties, its low molecular weight, high surface activity, and enhanced molecular mobility. These findings align with Hosseiniyeh et al. (2024), who observed higher EE in lecithin‐based black cumin EONE (84.28%) compared to whey protein–stabilized formulations (61.30%).

### 3.8 Optimal Nanoemulsion Based on GSO and Co‐Emulsifier Concentrations

The samples containing 6% LGEO and SL, without GSO and Tween 80 (sample 1), and also 6% LGEO, SL, 1.5% GSO, and 1.5% Tween 80 (sample 2) were selected as optimal NE samples of the second stage based on small particle size, PDI, high zeta potential, and EE results for fortification of orange juice at different concentrations (0%, 0.2%, 0.4%, and 0.6% w/w).

### 3.9 Morphology of the Optimum Nanoemulsions

**FIGURE 6:**
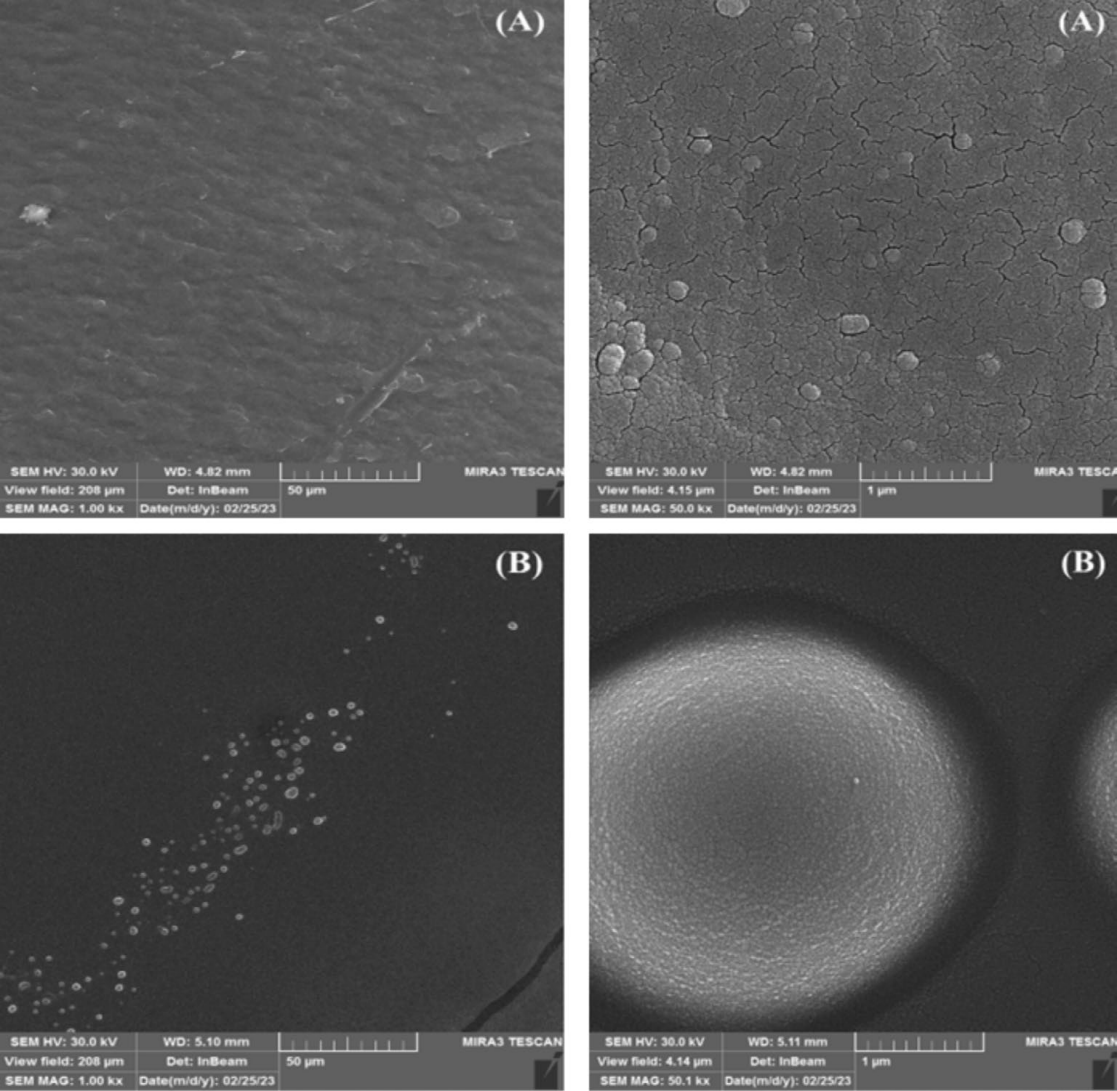
SEM micrographs of optimum NEs (A) 6% EO and 6% SL (B) 6% EO, 6% SL, 1.5% GSO, 1.5% Tween 80.

The SEM images of the NE samples of LGEO based on GSO are presented in Figure 6. As seen from the images related to sample NE 1, the NE particles were spherical in shape and irregular, with small cracks and fissures observed on the surface of the NE. In contrast, in NE 2, no cracks or irregularities were detected, and the particles appeared to be spherical. This condition can be attributed to the presence of Tween 80, as it produces a NE with a uniform state and orderly spherical particles, while lecithin is capable of creating an irregular state with cracks and fissures on the surface and irregular particles in the NE (Kaur et al. 2021). According to Zhao et al. (2017), NEs with spherical particles easily penetrate into cells. The obtained results are consistent with those of Kaur et al. (2021) for cinnamon EONEs, where the samples produced with Tween 80 exhibited a uniform state with spherical particles, while those produced with SL showed an irregular state with uneven particles.

### 3.10 FTIR

**FIGURE 7:**
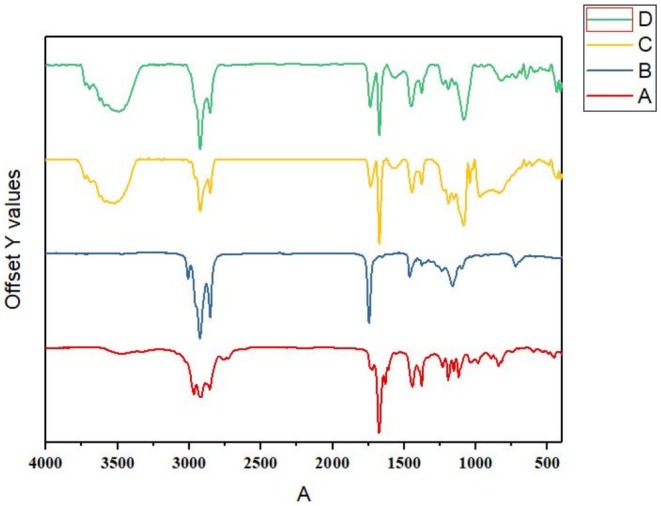
FTIR spectra of (A) LGEO, (B) GSO, (C) NE 1 (6% EO and 6% SL), (D) NE 2 (6% EO, 6% SL, 1.5% GSO, and 1.5% Tween 80).

FTIR spectroscopy was used to analyze chemical interactions in the samples. Figure 7 presents the FTIR spectra of the LGEO (A), GSO (B), NE 1 (C), and NE 2 (D). In the spectrum corresponding to the LGEO (A), a broad peak around 3482 cm^−1^ is likely attributed to OH stretching vibration. Significant absorption bands at 2968, 2918, 2857, and 2760 cm^−1^ are associated with the stretching vibrations of CH bonds in sp^3^ alkanes. The peaks observed at 1724, 1676, and 1632 cm^−1^ may be linked to the presence of carbonyl C=O vibrational groups in aldehydes and ketones found in the EO. Generally, the presence of high‐intensity peaks indicates a substantial number of aldehydes in the LGEO (Alipanah et al. 2022; Olayemi et al. 2018; Ranjbar et al. 2023). Another characteristic peak at 1443 cm^−1^ signifies the presence of a C=C stretching bond resulting from an aromatic compound in the EO. The absorption bond at 1378 cm^−1^ is due to CH_3_ variations. The absorption band at 1232 cm^−1^ corresponds to C–N vibrations resulting from amines, and the peaks at 1194, 1153, and 112 cm^−1^ are related to C–O stretching vibrations, indicating the presence of alcohols, ethers, and esters in the LGEO. The peaks at 986 and 842 cm^−1^ are associated with symmetric C–H absorption and C–H vibrational modes in the benzene ring of the EO, respectively (Olayemi et al. 2018).

The FTIR spectrum of the GSO is shown in Figure 7B. The peak formed at a wavenumber of 3008 cm^−1^ is likely related to the stretching vibrations of =C–H in the cis configuration. Characteristic peaks in the range of 2925 and 2854 cm^−1^ are attributed to the symmetric and asymmetric stretching vibrations of methylene (CH_2_) and methyl (CH_3_) groups associated with aliphatic triglycerides. The absorption peak at 1746 cm^−1^ is associated with the stretching vibrations of carbonyl (C=O) groups present in esters (Akin et al. 2019). The peak observed at 1463 cm^−1^ is attributed to the bending vibrations of CH_3_ and CH_2_ in aliphatic groups. The absorption bands at 1377 and 1238 cm^−1^, respectively, indicate the presence of bending and symmetric vibrations of CH_3_ and stretching vibrations of C–O in esters (Berezin et al. 2018). The peak at 1163 cm^−1^ relates to changes in the vibrational bonds of C–H in fatty acids, while the peak at 722 cm^−1^ is due to the stretching vibrations of C–O and C–C groups (Vladimír et al. 2021).

In comparing the FTIR spectra of NE sample 1 (Figure 7C) and NE type 2 (Figure 7D) with the spectra of LGEO and GSO, it can be observed that no new peaks were generated in the spectra of the NE samples. The results demonstrate the successful emulsification of LGEO by SL, GSO, and Tween 80, as evidenced by the preservation of characteristic LGEO and GSO peaks in the NE spectra. The observed interactions appear to be primarily electrostatic in nature, as indicated by the maintenance of molecular signatures without the formation of new covalent bonds. This suggests that the stabilization mechanism involves physical interactions rather than chemical bonding between components. The electrostatic interactions help to preserve the natural characteristics of the bioactive compounds in the EO, thus maintaining its antioxidant and antimicrobial properties (Karimi Sani et al. 2019; Xue et al. 2018).

### 3.11 Antimicrobial Activity of the Nanoemulsions

**FIGURE 8:**
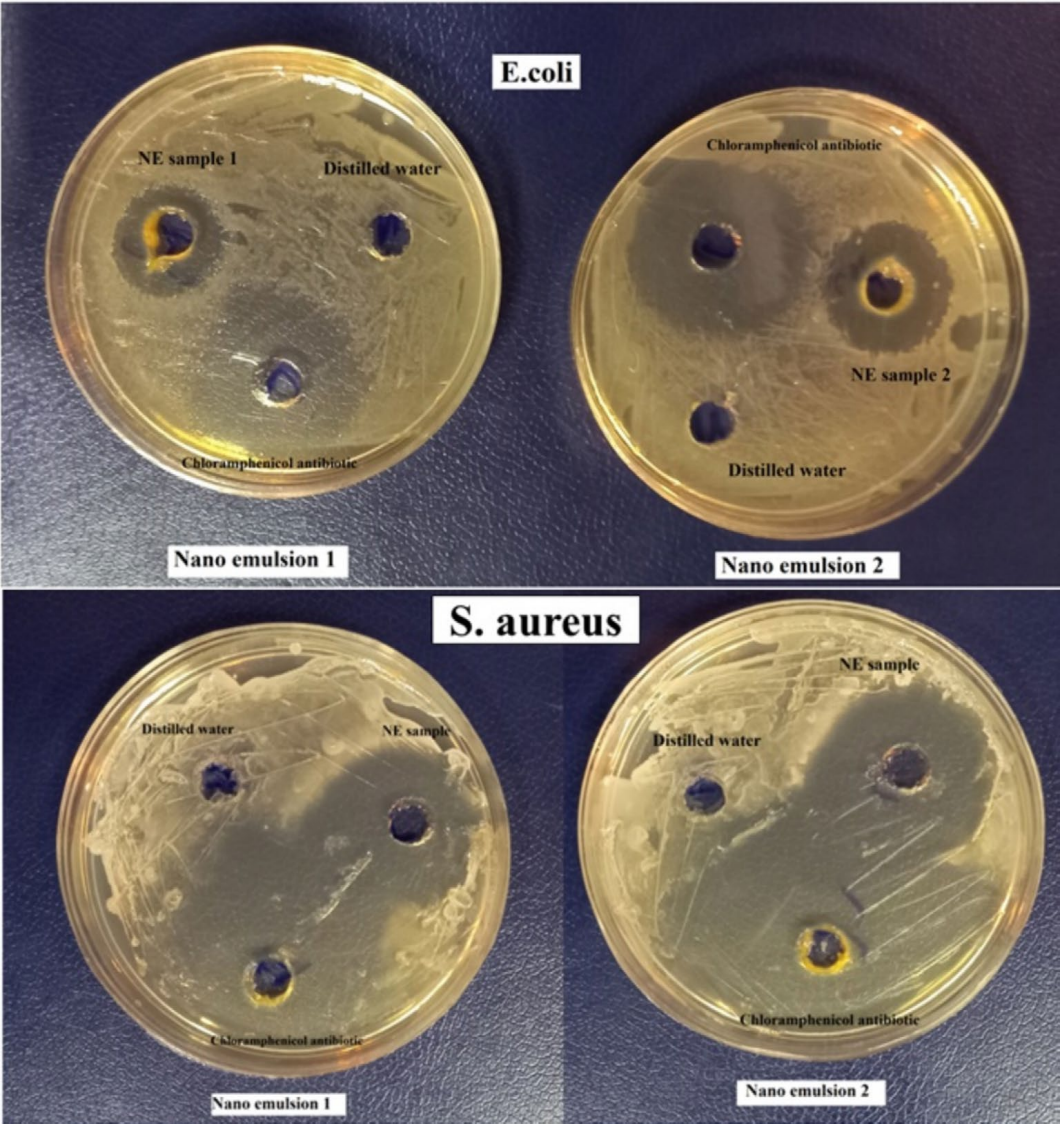
Antimicrobial effect of NE, chloramphenicol antibiotic, and distilled water. NE 1: 6% EO and SL, 0% GSO, 0% Tween 80; NE 2: 6% EO and SL, 1.5% GSO, 1.5% Tween 80.

The images showing the inhibition zone due to the antimicrobial properties of the LGEO nanoemulsion are presented in Figure 8, where the antimicrobial properties of the LGEO nanoemulsion were compared with the strong antibiotic chloramphenicol and a control sample of distilled water. Generally, the larger the diameter of the inhibition zone, the greater the antimicrobial property (Franklyne et al. 2019; Zhang et al. 2017). The results showed that the inhibitory effect of the antibiotic chloramphenicol was higher compared to both types of NEs, resulting in a larger inhibition zone. Additionally, no antimicrobial properties were observed in the control sample of distilled water. Furthermore, the inhibitory effect in both selected optimized NEs against *Staphylococcus aureus* ( *S. aureus* ) (a Gram‐positive bacterium) was greater than that against *Escherichia coli* ( *E. coli* ) (a Gram‐negative bacterium), which is due to the greater sensitivity of Gram‐positive bacteria to external agents like EOs and extracts compared to Gram‐negative bacteria. This sensitivity arises from differences in their cell wall structures, as Gram‐negative bacteria have an outer layer composed of a phospholipid and protein membrane and a peptidoglycan layer (Azhdarzadeh and Hojjati 2016; Liew et al. 2020). The antimicrobial properties of the NE are attributed to the presence of bioactive compounds with antimicrobial activity, such as citral, alpha‐pinene, and limonene, which disrupt and penetrate the bacterial cell walls, leading to cell membrane damage, denaturation of cellular proteins, cytoplasmic leakage, and ultimately bacterial death. In a study, Yazgan et al. (2019) found that the antimicrobial properties of lemon EO were greater against *S. aureus* . According to Yildirim et al. (2017), the antimicrobial properties of the NE are related to the compounds used in its formulation. Additionally, based on the results, the antimicrobial properties of NE 2 were greater than those of NE 1, likely due to the presence of GSO in the formulation of this NE. The findings align with the results of Liew et al. (2020) for lemon EONE, which reported greater sensitivity to Gram‐positive bacteria compared to Gram‐negative bacteria. Moreover, Liu et al. (2021) noted the higher resistance of the Gram‐negative bacterium *E. coli* compared to the Gram‐positive *S. aureus* for cinnamon NE EO, and Seibert et al. (2019) reported similar results for propolis NE.

### 3.12 Release of EO From Nanoemulsions

**FIGURE 9:**
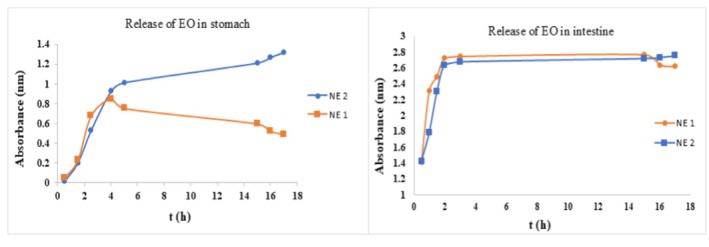
Release of EO of NE 1 (6% EO and SL, 0% GSO, 0% Tween 80) and NE 2 (6% EO and SL, 1.5% GSO, 1.5% Tween 80) in stomach and intestine condition.

The NE of LGEO needs to pass through the stomach and enter the intestine to act as an antimicrobial compound, preservative, and flavoring agent. The release profile of EOs from optimized NEs was evaluated under simulated gastrointestinal conditions (pH 1.2 ± 0.1 and 7.4 ± 0.1), as shown in Figure 9. In the simulated gastric conditions with the presence of the enzyme pepsin, the release of EO from the dialysis bag into the buffer medium initially increased over time for both samples. The release rate in NE 1 (6% EO and SL, 0% GSO, and Tween 80) decreased after the initial increase and then stabilized. In contrast, for NE sample 2 (6% EO and lecithin, 1.5% GSO, and Tween 80), no decrease in the amount of released EO was observed, and it remained constant after the initial increase. The release rate in NE sample 1 was higher compared to sample 2. The slower release rate in sample 2 is due to the presence of GSO and Tween 80, which is due to the slow breakage of the NE droplets. In other words, Tween 80 protects the EO during the digestive process in the early hours. In the simulated intestinal digestion, the release of EO from the remaining NEs sharply increased. This sudden increase is due to the presence of bile salts as an emulsifying compound that can release the EO from the emulsified capsules (Hou et al. 2021). Additionally, the higher release rate in the simulated intestinal environment at pH 7.4 ± 0.1 compared to the gastric environment at pH 1.2 ± 0.1 indicates a greater sensitivity of the NE to pH, which relates to the interactions and factors influencing release in both alkaline and acidic environments. Under the simulated intestinal conditions in the presence of the enzyme pancreatin, the release amount increased over time to a certain extent for both samples and then stabilized. The release rate in NE 1 remained higher than in sample 2. The presence of Tween 80 in NE 2, similar to its effect in the gastric environment, hinders the breakage of NE droplets and the rapid release of the EO (Fahami and Fathi 2018). According to Timilsena et al. (2017), the release of oil from chia seeds combined with gum and protein was greater under simulated gastric conditions, which was attributed to the enzymatic degradation of protein during the digestion process.

### 3.13 Antioxidant Properties

Antioxidant activity assessment using the DPPH radical scavenging method revealed significant differences between free LGEO and the optimized NEs (*p* < 0.05). As presented in Table 5, free LGEO demonstrated superior antioxidant activity (75.20% ± 0.02%) compared to the NEs (32.72% ± 0.01% and 43.14% ± 0.01%). The higher antioxidant activity in the free form of EO compared to the NE may be associated with a greater presence of phenolic and flavonoid compounds. The phenolic compounds found in LGEO, such as geraniol, neral, and citral, contribute to a high antioxidant capacity due to their high hydroxyl content. Additionally, in the free EO, phenolic compounds are directly accessible, whereas in the NE, these compounds are encapsulated within the NE droplets, controlling their release. According to a report by Perumal et al. (2021), the antioxidant activity of free green tea EO was greater than that of the NE form. Moreover, based on the obtained results, the antioxidant activity of NE 2 (43.14% ± 0.01%) was higher than NE 1 (32.72% ± 0.01%), likely due to the presence of antioxidant compounds in GSO and LGEO, whereas the antioxidant property in sample 1 is attributed to the compounds present in LGEO.

**TABLE 5:** Antioxidant activity of optimal NE and free EO.

Sample	Antioxidant activity (%)
NE 1	32.72 ± 0.01^a^
NE 2	43.14 ± 0.01^b^
Free EO	75.20 ± 0.02^c^

*Note:* NE 1: 6% EO and SL, 0% GSO, 0% Tween 80; NE 2: 6% EO and SL, 1.5% GSO, 1.5% Tween 80. The results are given as mean ± standard deviation. Different small letters represent a significant difference (*p* ≤ 0.05) per column.

### 3.14 Orange Juice Attributes

**FIGURE 10:**
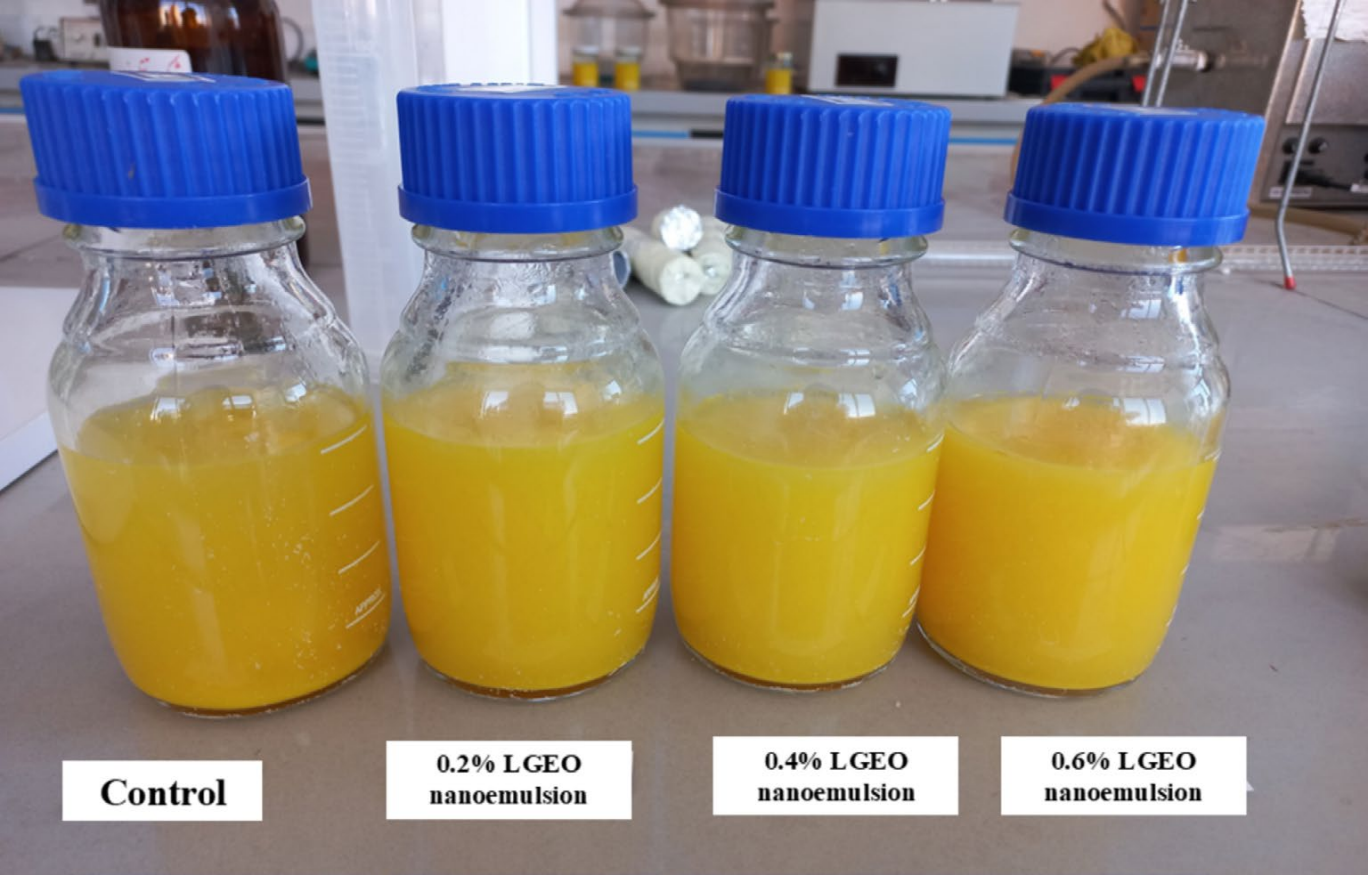
Image of prepared orange juice containing (0%, 0.2%, 0.4%, and 0.6%) LGEO nanoemulsion at the first day.

As represented in Figure 10, orange juice samples containing 0%, 0.2%, 0.4%, and 0.6% optimal LGEO nanoemulsion were prepared. Antimicrobial and chemical properties and sensorial analysis of these samples were done during the storage time (1st, 15th, and 45th days).

#### 3.14.1 Antioxidant Activity

As shown in Table 6, the antioxidant activity of the fruit juice incorporated with both types of LGEO nanoemulsion (0.2%–0.6%) increased significantly compared to the control (*p* < 0.05). It can be attributed to the presence of surfactants surrounding the NE droplets. These surfactants not only protect the phenolic compounds and the functional properties of the EO but also enhance the oil's solubility in aqueous environments (Dong et al. 2019). The antioxidant capacity of the juice samples was enhanced by phenolic compounds present in LGEO, particularly citral, geraniol, limonene, and neral. Comparative analysis revealed that samples containing NE 1 demonstrated significantly higher antioxidant activity than those with NE 2 (*p* < 0.05). Furthermore, all orange juice formulations showed a progressive increase in antioxidant activity throughout the 30‐day storage period. The increase in antioxidant properties during storage can be attributed to the elevation of bioactive compounds, such as phenolic compounds and flavonoids, which are known as potent antioxidants and natural fungicides (Cenobio‐Galindo et al. 2019). Additionally, the increase in antioxidant activity may be related to phenolic compounds in EO, which become more available or better dispersed in the juice matrix over time. Similar findings were reported by Ishkeh et al. (2021), who stated that during the 9‐day shelf life of coated raspberries with chitosan NE, an increase in antioxidant properties was observed over time.

**TABLE 6:** The antioxidant activity of orange juice containing LGEO nanoemulsion during storage time.

Sample	Day 0	Day 15	Day 30
Control	14.38 ± 0.68^aA^	48.63 ± 0.61^aB^	54.21 ± 0.11^aC^
0.2% NE 1	44.20 ± 0.15^cA^	55.73 ± 0.54^bB^	64.82 ± 0.81^bC^
0.4% NE 1	64.07 ± 0.38^dA^	68.16 ± 0.23^dC^	66.19 ± 0.71^bB^
0.6% NE 1	77.38 ± 0.46^fB^	68.03 ± 0.73^dA^	68.97 ± 0.40^cA^
0.2% NE 2	22.30 ± 0.93^bA^	55.80 ± 0.42^bB^	66.59 ± 0.71^bC^
0.4% NE 2	42.27 ± 0.38^cA^	62.63 ± 0.94^cB^	66.12 ± 0.65^bB^
0.6% NE 2	61.68 ± 0.72^eA^	72.61 ± 0.51^eB^	72.58 ± 0.40^dB^

*Note:* NE 1: 6% EO and SL, 0% GSO, 0% Tween 80; NE 2: 6% EO and SL, 1.5% GSO, 1.5% Tween 80. The results are given as mean ± standard deviation. Different small letters represent a significant difference (*p* ≤ 0.05) per column. Different capital letters represent a significant difference (*p* ≤ 0.05) per row.

#### 3.14.2 Acidity

Table 7 shows significant differences in acidity among orange juice samples containing lemon EONE across the study period (*p* < 0.05). The control sample consistently exhibited the lowest acidity levels, while all NE‐fortified samples demonstrated significantly higher acidity throughout the experimental duration. This behavior may be related to acidic compounds in LGEO, so they can increase the acidity of the orange juice. These compounds can be transferred to the juice. According to the results, over a storage time of up to 30 days, the acidity of the orange juice samples containing lemon EONE significantly increased (*p* < 0.05) (Table 7). The increase in acidity over the storage period is due to the conversion of acids into sugars and salts by enzymes, especially invertase. Moreover, microbial activity can also lead to an increase in acidity (Shamsudin et al. 2020). Consistent results regarding the increase in acidity and decrease in pH were presented by Radi et al. (2018), who stated that the addition of orange peel NE EO to freshly cut orange fruit yielded similar results over time.

**TABLE 7:** The acidity of orange juice containing LGEO nanoemulsion during storage time.

	Acidity (g of citric acid/100 mL)		
	0	15	30
Control	17.53 ± 0.05^aA^	23.5 ± 0.09^aB^	23.63 ± 0.02^aB^
0.2% NE 1	21.76 ± 0.05^bA^	23.64 ± 0.09^aB^	23.77 ± 0.01^bB^
0.4% NE 1	21.88 ± 0.1^bcA^	24.26 ± 0.02^bB^	24.59 ± 0.02^cB^
0.6% NE 1	21.91 ± 0.09^cA^	24.8 ± 0.02^cB^	24.91 ± 0.06^bcB^
0.2% NE 2	22.73 ± 0.05^dA^	24.95 ± 0.05^cB^	24.98 ± 0.01^cdB^
0.4% NE 2	22.77 ± 0.05^deA^	25.01 ± 0.01^cB^	25.14 ± 0.05^cdB^
0.6% NE 2	22.88 ± 0.02^eA^	25.07 ± 0.01^cB^	25.29 ± 0.03^eB^

*Note:* NE 1: 6% EO and SL, 0% GSO, 0% Tween 80; NE 2: 6% EO and SL, 1.5% GSO, 1.5% Tween 80. The results are given as mean ± standard deviation. Different small letters represent a significant difference (*p* ≤ 0.05) per column. Different capital letters represent a significant difference (*p* ≤ 0.05) per row.

#### 3.14.3 Antimicrobial Activity

**FIGURE 11:**
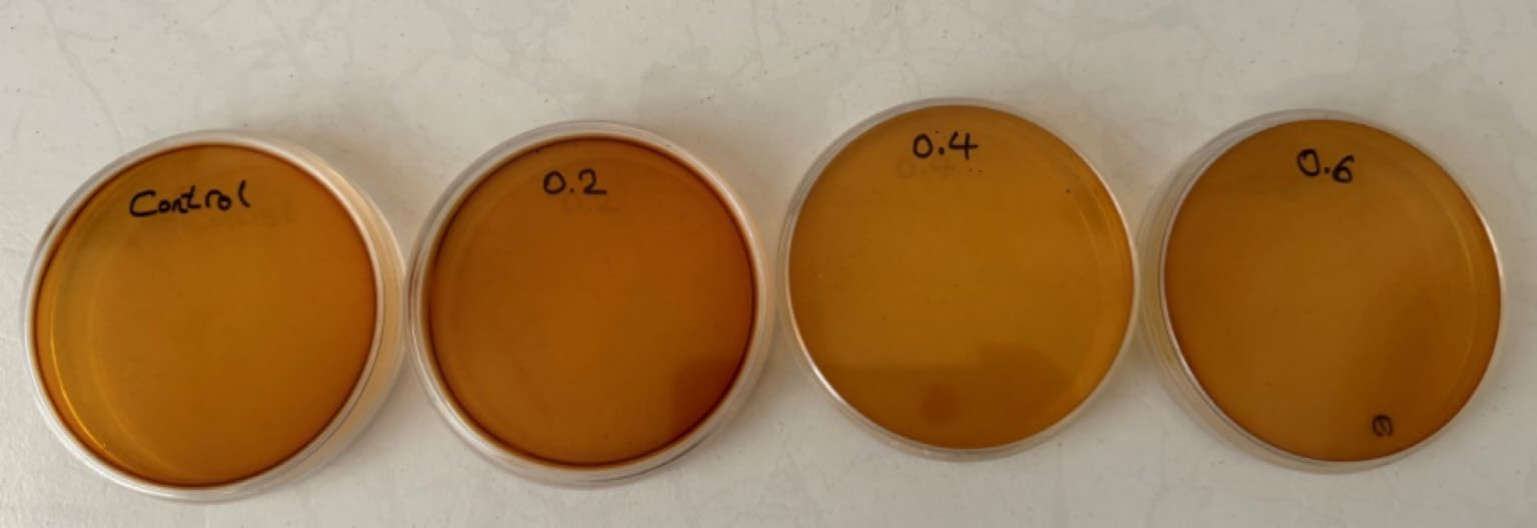
Count of yeast and mold of optimum NEs on the 30th day. NE 1: 6% EO and SL, 0% GSO, 0% Tween 80; NE 2: 6% EO and SL, 1.5% GSO, 1.5% Tween 80.

The findings from the total microbial count, mold, and yeast assessments in the orange juice samples enriched with different concentrations of NE of LGEO are illustrated in Figure 11 and detailed in Table 8. Notably, no mold or yeast contamination was detected in the orange juice samples containing different levels of NE types 1 and 2 throughout the 30‐day storage. These results confirmed the absence of microbial contamination in both the orange juice samples and the produced NEs. Moreover, an increase in the concentration of the LGEO nanoemulsion led to a significant reduction in bacterial counts within the orange juice samples, with the control sample exhibiting the highest bacterial load. This observation highlights the favorable impact of incorporating NE on the total microbial load, which effectively delayed microbial growth. The observed decrease in microbial load may be attributed to the protective characteristics of the EO, which is consistently released from the NE. The antimicrobial activity of LGEO can be ascribed to the presence of terpenoids, such as citral, geraniol, and neral. Furthermore, over the course of 15–30 days of storage, there was a general increase in bacterial counts across all samples. Overall, the orange juices containing NE exhibited lower microbial loads, likely due to the hydrophobic compounds present in the LGEO that facilitate access to the cell walls and membranes of the bacteria in the orange juice. This mechanism leads to enhanced membrane permeability, the efflux of intracellular materials, and ultimately, microbial cell death, as supported by Gheybi et al. (2024). The antimicrobial efficacy of LGEO nanoemulsions may increase progressively during storage due to the sustained release and enhanced bioavailability of volatile and phenolic compounds. This time‐dependent mechanism explains the observed gradual reduction in microbial load in orange juice samples containing LGEO nanoemulsions (Gao et al. 2020). Zhou et al. (2021) also reported that increasing the concentration of galangal EO in pineapple juice resulted in a reduction in microbial load, while prolonged storage time contributed to an increase in microbial populations in the samples. Additionally, other research by Radi et al. (2017) indicated that the incorporation of NE derived from orange peel EO resulted in elevated microbial loads in sliced orange fruit over time.

**TABLE 8:** Total microbial count of orange juice containing LGEO nanoemulsion during storage time.

Sample	Day 0	Day 15	Day 30
Control	5.13 ± 0.04^cA^	5.43 ± 0.03^eB^	5.55 ± 0.01^eC^
0.2% NE 1	4.85 ± 0.13^bA^	5.16 ± 0.10^deB^	5.34 ± 0.04^dC^
0.4% NE 1	4.25 ± 0.27^Aa^	5.04 ± 0.11^cdB^	5.14 ± 0.05^cC^
0.6% NE 1	4.10 ± 0.17^aA^	4.56 ± 0.24^Bb^	4.79 ± 0.10^bC^
0.2% NE 2	4.93 ± 0.02^bA^	5.15 ± 0.10^Db^	5.40 ± 0.07^dC^
0.4% NE 2	4.69 ± 0.08^bA^	4.80 ± 0.18^bcB^	5.08 ± 0.05^cC^
0.6% NE 2	4.10 ± 0.17^aA^	4.25 ± 0.24^aB^	4.51 ± 0.07^aC^

*Note:* NE 1: 6% EO and SL, 0% GSO, 0% Tween 80; NE 2: 6% EO and SL, 1.5% GSO, 1.5% Tween 80. The results are given as mean ± standard deviation. Different small letters represent a significant difference (*p* ≤ 0.05) per column. Different capital letters represent a significant difference (*p* ≤ 0.05) per row.

#### 3.14.4 Sensorial Attributes

The sensory evaluation results of the orange juice samples containing NEs for color, taste, aroma, and overall acceptance are presented in Table 9. Based on the analysis of variance among the orange juice samples on days 1, 15, and 30 after storage, there were no significant differences in color and taste characteristics. According to the evaluators' assessments, the control sample received the highest scores for aroma and overall acceptance on the first day of storage. The orange juice sample containing 0.2% of NE 1 had the lowest aroma score. Additionally, the lowest overall acceptance in the enriched samples was observed for the juice containing 0.6% of NE 1. The results showed that, on the 15th day of storage, the control sample and the sample enriched with 0.2% of emulsified EO type 1 received the lowest and highest aroma scores, respectively. On the same day, the sample containing 0.6% of NE 2 had the lowest desirability according to the panelists, while the highest desirability was attributed to the sample containing 0.2% of NE 1. On the 30th day of storage, the orange juice samples enriched with 0.6% and 0.4% of NE 2 received the lowest and highest scores for taste and overall acceptance, respectively. The results from the sensory assessment of the samples over time showed that until the 15th day of storage, the scores for taste and overall acceptance increased but then decreased on the 30th day of storage. Furthermore, the taste of the samples showed a downward trend over the storage period. Generally, the sensory results indicated that the orange juice samples had the best characteristics on the 15th day of storage.

**TABLE 9:** Sensory analysis of orange juice samples containing LGEO nanoemulsion during storage time.

Sample	Color	Taste	Odor	Overall acceptance
Day 0				
Control	4.5 ± 0.7^a^	3.6 ± 0.8^a^	4.5 ± 0.70^d^	4.0 ± 0.6^d^
0.2% NE 1	4.2 ± 0.7^a^	3.4 ± 0.1^a^	3.4 ± 1.26^a^	3.7 ± 1.0^c^
0.4% NE 1	4.3 ± 0.8^a^	3.2 ± 0.6^a^	3.5 ± 1.08^a^	3.3 ± 0.4^b^
0.6% NE 1	4.8 ± 0.4^a^	2.5 ± 0.7^a^	3.8 ± 1.13^b^	2.9 ± 0.7^a^
0.2% NE 2	4.7 ± 0.4^a^	3.4 ± 0.8^a^	4.2 ± 0.91^c^	3.7 ± 0.8^c^
0.4% NE 2	4.7 ± 0.6^a^	3.1 ± 0.9^a^	4.2 ± 1.13^c^	3.3 ± 0.4^b^
0.6% NE 2	4.3 ± 0.4^a^	3.0 ± 0.9^a^	3.9 ± 0.87^b^	3.2 ± 0.9^b^
Day 15				
Control	4.8 ± 0.4^a^	3.3 ± 1.25^a^	2.9 ± 1.2^a^	3.7 ± 0.6^c^
0.2% NE 1	4.7 ± 0.6^a^	3.8 ± 1.39^a^	4.4 ± 0.9^d^	4.1 ± 1.1^c^
0.4% NE 1	4.7 ± 0.6^a^	3.3 ± 1.56^a^	3.7 ± 0.8^b^	3.4 ± 1.0^b^
0.6% NE 1	4.7 ± 0.6^a^	3.3 ± 1.33^a^	4.1 ± 0.8^cd^	3.8 ± 1.2^c^
0.2% NE 2	4.7 ± 0.4^a^	3.4 ± 1.07^a^	4.2 ± 0.7^cd^	3.9 ± 0.7^c^
0.4% NE 2	4.7 ± 0.6^a^	3.6 ± 1.07^a^	4.0 ± 0.9^bc^	4.0 ± 1.1^c^
0.6% NE 2	4.6 ± 0.5^a^	2.5 ± 1.26^a^	3.9 ± 0.8^bc^	3.1 ± 1.1^a^
Day 30				
Control	4.8 ± 0.4^a^	3.1 ± 1.1^c^	3.1 ± 0.7^c^	3.4 ± 1.0^c^
0.2% NE 1	4.9 ± 0.3^a^	3.5 ± 1.0^d^	3.5 ± 0.5^d^	4.2 ± 0.7^d^
0.4% NE 1	4.8 ± 0.4^a^	3.6 ± 0.5^d^	3.6 ± 0.5^d^	3.7 ± 0.8^c^
0.6% NE 1	4.8 ± 0.4^a^	2.7 ± 0.6^b^	2.7 ± 0.5^b^	2.9 ± 0.7^b^
0.2% NE 2	4.6 ± 0.5^a^	2.7 ± 0.6^b^	2.7 ± 0.7^b^	3.6 ± 0.6^c^
0.4% NE 2	4.8 ± 0.4^a^	3.8 ± 0.6^e^	3.8 ± 0.7^e^	4.3 ± 0.6^d^
0.6% NE 2	4.7 ± 0.4^a^	1.3 ± 0.4^a^	1.3 ± 0.6^a^	2.3 ± 0.4^a^

*Note:* NE 1: 6% EO and SL, 0% GSO, 0% Tween 80; NE 2: 6% EO and SL, 1.5% GSO, 1.5% Tween 80. The results are given as mean ± standard deviation. Different small letters represent a significant difference (*p* ≤ 0.05) per column. Different capital letters represent a significant difference (*p* ≤ 0.05) per row.

## 4 Conclusions

Orange juice is one of the healthiest and most widely consumed fruit juices worldwide, but it is susceptible to microbial spoilage. One effective way to protect fruit juice from spoilage is by using antioxidant and antimicrobial compounds such as EOs. EOs are natural compounds containing bioactive molecules with antimicrobial, antioxidant, anti‐allergenic, and anticancer properties, making them suitable for minimizing microbial spoilage and extending the shelf life of food products. Nano‐emulsification helps preserve aroma and flavor, enhance stability, maintain nutritional properties, and increase the bioavailability of EOs. Enriching orange juice with nano‐emulsified EO is a novel method to improve its functional and nutritional properties. The results from the release rate under simulated gastrointestinal conditions indicated that the effective compounds of the nano‐emulsified EO were preserved under the low pH of the stomach (gastric acidity). Moreover, a controlled release behavior was observed for the EOs and active compounds during the digestion process. The acidity level also showed an upward trend over time. The presence of nano‐emulsified EO in orange juice prevented the growth of molds and yeasts in the samples. Overall acceptability, taste, and aroma in the orange juice samples were maximized on the 15th day of storage. In conclusion, the findings of this study suggest that nano‐emulsified LGEO can be used for fortifying food products and increasing their shelf life due to its promising antioxidant and antimicrobial properties.

## Author Contributions

**Mahsa Nouraddini:** conceptualization (equal), funding acquisition (equal), methodology (equal), resources (equal), visualization (equal), writing – original draft (equal). **Forogh Mohtarami:** data curation (equal), formal analysis (equal), investigation (equal), methodology (equal), software (equal), writing – review and editing (equal). **Mohsen Esmaiili:** conceptualization (equal), funding acquisition (equal), investigation (equal), methodology (equal), project administration (equal), supervision (equal), validation (equal), visualization (equal), writing – review and editing (equal).

## Ethics Statement

The authors have nothing to report.

## Conflicts of Interest

The authors declare no conflicts of interest.

## Data Availability

Data will be available on request. If someone wants to request the data from this study, they should be in contact with Ms. Mahsa Nouraddini (mahsa.noradini@gmail.com).
